# Insomnia-related brain functional correlates in first-episode drug-naïve major depressive disorder revealed by resting-state fMRI

**DOI:** 10.3389/fnins.2024.1290345

**Published:** 2024-08-29

**Authors:** Ke Dai, Xianwei Liu, Jun Hu, Fangfang Ren, Zhuma Jin, Shulan Xu, Ping Cao

**Affiliations:** ^1^Department of Radiology, Nanjing Brain Hospital, Affiliated Hospital of Nanjing Medical University, Nanjing, China; ^2^Department of Psychiatry, Nanjing Brain Hospital, Affiliated Hospital of Nanjing Medical University, Nanjing, China; ^3^Department of Gerontology, Nanjing Brain Hospital, Affiliated Hospital of Nanjing Medical University, Nanjing, China

**Keywords:** major depressive disorder, insomnia, resting-state functional magnetic resonance imaging, regional homogeneity, insula

## Abstract

**Introduction:**

Insomnia is a common comorbidity symptom in major depressive disorder (MDD) patients. Abnormal brain activities have been observed in both MDD and insomnia patients, however, the central pathological mechanisms underlying the co-occurrence of insomnia in MDD patients are still unclear. This study aimed to explore the differences of spontaneous brain activity between MDD patients with and without insomnia, as well as patients with different level of insomnia.

**Methods:**

A total of 88 first-episode drug-naïve MDD patients including 44 with insomnia (22 with high insomnia and 22 with low insomnia) and 44 without insomnia, as well as 44 healthy controls (HC), were enrolled in this study. The level of depression and insomnia were evaluated by HAMD-17, adjusted HAMD-17 and its sleep disturbance subscale in all subjects. Resting-state functional and structural magnetic resonance imaging data were acquired from all participants and then were preprocessed by the software of DPASF. Regional homogeneity (ReHo) values of brain regions were calculated by the software of REST and were compared. Finally, receiver operating characteristic (ROC) curves were conducted to determine the values of abnormal brain regions for identifying MDD patients with insomnia and evaluating the severity of insomnia.

**Results:**

Analysis of variance showed that there were significant differences in ReHo values in the left middle frontal gyrus, left pallidum, right superior frontal gyrus, right medial superior frontal gyrus and right rectus gyrus among three groups. Compared with HC, MDD patients with insomnia showed increased ReHo values in the medial superior frontal gyrus, middle frontal gyrus, triangular inferior frontal gyrus, calcarine fissure and right medial superior frontal gyrus, medial orbital superior frontal gyrus, as well as decreased ReHo values in the left middle occipital gyrus, pallidum and right superior temporal gyrus, inferior temporal gyrus, middle cingulate gyrus, hippocampus, putamen. MDD patients without insomnia demonstrated increased ReHo values in the left middle frontal gyrus, orbital middle frontal gyrus, anterior cingulate gyrus and right triangular inferior frontal gyrus, as well as decreased ReHo values in the left rectus gyrus, postcentral gyrus and right rectus gyrus, fusiform gyrus, pallidum. In addition, MDD patients with insomnia had decreased ReHo values in the left insula when compared to those without insomnia. Moreover, MDD patients with high insomnia exhibited increased ReHo values in the right middle temporal gyrus, and decreased ReHo values in the left orbital superior frontal gyrus, lingual gyrus, right inferior parietal gyrus and postcentral gyrus compared to those with low insomnia. ROC analysis demonstrated that impaired brain region might be helpful for identifying MDD patients with insomnia and evaluating the severity of insomnia.

**Conclusion:**

These findings suggested that MDD patients with insomnia had wider abnormalities of brain activities in the prefrontal-limbic circuits including increased activities in the prefrontal cortex, which might be the compensatory mechanism underlying insomnia in MDD. In addition, decreased activity of left insula might be associated with the occurrence of insomnia in MDD patients and decreased activities of the frontal–parietal network might cause more serious insomnia related to MDD.

## 1 Introduction

Major depressive disorder (MDD) is a common and debilitating psychiatric disorder, which is characterized by different clinical manifestations including depressed mood, decreased or loss of interest and pleasure, cognitive impairment in different domains, along with vegetative symptoms, such as sleep disturbances (Otte et al., 2016). MDD affects about 12% of the adult population globally with rate about twice as high in women than in men (Seedat et al., 2009; Bromet et al., 2011; Hasin et al., 2018). It is approximately estimated that 55% of MDD patients experience suicidal ideation leading a higher risk of suicide and up to 50% of suicides occur during depressive episodes per year worldwide (Sokero et al., 2003). MDD patients are 20–30 times more likely to die by suicide than the general population (Chesney et al., 2014). In addition, MDD is considered as the second largest contributor to the burden of chronic diseases and it is associated with an increased risk of developing other medical conditions, further increasing the burden of MDD (GBD 2016 DALYs and HALE Collaborators, 2017). Sleep disturbances, particularly insomnia, are prevalent in MDD patients and 92% of MDD patients reported substantial sleep complaints (Geoffroy et al., 2018). Insomnia can be a common condition independent of depression affecting approximately 10% of the general population, which causes higher medical costs and impaired work performance (National Institutes of Health, 2005). Insomnia, one of the main manifestations of sleep disturbances, is considered as the prodromal clinical characteristic of MDD and it is recommended as a diagnostic characteristic of MDD (Perlis et al., 1997). Individual with insomnia often have higher risk for developing MDD and insomnia is an important risk factor for the recurrence of MDD (Baglioni et al., 2011; Li et al., 2012). In addition, insomnia is related to the severity and therapeutic effect of MDD, as well as the risk of suicide and quality of life of patients (McCall et al., 2000; Jindal and Thase, 2004; Howland, 2011; Richardson et al., 2017; Pompili et al., 2013). MDD patients comorbid with insomnia, a common residual symptom of MDD, often have negative outcomes including incomplete treatment response, higher rates of relapse and increased medical morbidity (Nierenberg et al., 1999; Nelson et al., 2005; Léger et al., 2002). Insomnia frequently co-occurs with MDD and it can persist into the remission stage of MDD, or even after remission (Tsuno et al., 2005).

MDD is a clinically and biologically heterogeneous syndrome. The relationships between MDD and insomnia are bidirectional. Recognizing discrete subtypes of MDD with distinguishing neurobiological substrates and clinical features, as well as analyzing specific symptoms and their causal relationships, might be promising strategies for guiding personalized treatment of MDD (Wang et al., 2021). Therefore, it is worthwhile to explore the central neural mechanisms of MDD-related sleep disorders. Exploring the mechanisms underlying functional brain abnormalities associated with insomnia in MDD patients may be helpful for developing individualized therapies for MDD patients with insomnia. However, the brain functional correlates associated with insomnia and MDD have been explored extensively as separate entities and the central neural mechanisms underlying insomnia and MDD comorbidity are unclear (Yuan et al., 2022; Reimann et al., 2023).

Resting-state functional magnetic resonance imaging (rs-fMRI) is a non-invasive imaging technique to measure the spontaneous activity of human brain during rest based on blood oxygen level-dependent (BOLD) signal (Raimondo et al., 2021). It has been generally utilized to explore the physiological activity of human brain, as well as the pathological brain activity of psychiatric and neurological disorders (Mirzaei and Adeli, 2016; Long et al., 2020). Both structural and functional abnormalities have been identified in the brain of patients with MDD and insomnia by previous neuroimaging studies (Gray et al., 2020; Wu et al., 2020). MDD patients showed decreased functional connectivity within the frontoparietal network, which was composed of a set of regions involved in the cognitive control of emotion and attention (Kaiser et al., 2015). In addition, MDD patients had increased functional connectivity in the default mode network (DMN), which supported the emotional processing and internally oriented and self-referential thoughts (Kaiser et al., 2015). These findings suggested that MDD might be associated with the imbalanced functional connectivity between brain regions located in the control networks involved in mediating the top-down regulation of emotion and attention. In addition, abnormal spontaneous brain activities of emotion-related regions were found in insomnia patients and disrupted functional connectivity of frontoparietal network might be associated with the sleep quality and treatment response of insomnia patients (Zheng et al., 2023; Wang et al., 2016). Abnormalities of brain activities in the DMN and dorsal attention network were positively correlated with the severity of insomnia and these findings contributed to identify fMRI biomarkers for insomnia (Zhou et al., 2017).

MDD patients with different degrees of insomnia symptoms exhibited structural and functional disturbances in several brain regions, which included the amygdala, prefrontal cortex and anterior cingulate cortex and insula (Bagherzadeh-Azbari et al., 2019). In addition, the aberrant functional connectivity within and between the main hubs of the salience and DMN could potentially yield new insights into the link between MDD and insomnia (Bagherzadeh-Azbari et al., 2019). These findings suggested that neuroimaging methods could verily improve our understanding of the overlapping underlying neural mechanisms between MDD and insomnia, and advance the corresponding theories. Moreover, previous GWAS study demonstrated that the gene expression profiles of a few cortical and subcortical areas (including the caudate nucleus and Brodmann areas 9 and 24, striatum, claustrum, and hypothalamus) showed above-chance resemblance to the genetic risk profile of insomnia disorder, which suggested that these brain regions might be associated with the development of insomnia (Jansen et al., 2019). Based on amygdala-based resting-state functional connectivity (RSFC), increased RSFC in bilateral superior temporal gyrus, and decreased RSFC in left supplementary motor area and bilateral postcentral gyrus were identified in MDD patients with high insomnia when compared to patients with low insomnia (Ye et al., 2023). In addition, RSFC of the bilateral amygdala with superior temporal gyrus were positively related to the sleep disturbance scores. These findings suggested that RSFC in temporal lobe and other specifically activated regions might be associated with neural circuits involved with insomnia in MDD. Ample electrophysiological evidences have been accumulating regarding the pathological mechanisms in MDD (Shahaf, 2016). MDD patients, especially those with, often showed reduced attention Reduced P3 amplitude, delayed latency, or both (Gangadhar et al., 1993). Multiple regions and pathways, both cortical and sub-cortical, were considered to be involved in evoking P3 and attention (Shahaf and Pratt, 2013). Previous electrophysiology study proposed that sleep might be characterized by inadequate generation of restorative sleep in MDD patients, as indexed by reduced amounts of slow-wave activity (Cheng et al., 2015). Conversely, poor sleep might be resulted from intrusions of fast-frequency activity, which might be reflective of a hyperarousal central nervous system (Cheng et al., 2015). In this electrophysiology study, the findings suggested that MDD was characterized by an overall decrease in slow-wave activity, which was associated with elevated anxious and depressed mood the following morning (Cheng et al., 2015). Although previous studies evaluated functional abnormalities in patients with MDD and insomnia, the central pathological mechanisms underlying the co-occurrence of insomnia in MDD patients before drug treatment, especially first-episode drug-naïve MDD patients, are still unclear. Therefore, in order to eliminate the influence of drug treatments on brain activity, we only included first-episode drug-naïve MDD patients in the presented study.

In this study, we speculated that MDD patients with insomnia might have wider brain regions with abnormal brain activities, especially in the top-down circuits involved in the regulation of emotion compared to those without insomnia. In addition, patients with different level of insomnia might exhibit discrepant central pathophysiologic mechanisms. Therefore, we aimed to explore the differences of regional spontaneous brain activities during rest between first-episode drug-naïve MDD patients with insomnia (high insomnia and low insomnia) and without insomnia, as well as healthy controls (HC) with the measure of ReHo, which can evaluate the temporal synchronization between a given voxel and its neighboring voxels based on rs-fMRI data. Finally, receiver operating characteristic (ROC) curves were conducted to determine the values of abnormal brain regions for identifying MDD patients with insomnia and evaluating the severity of insomnia.

## 2 Materials and methods

### 2.1 Participants

**Figure 1:**
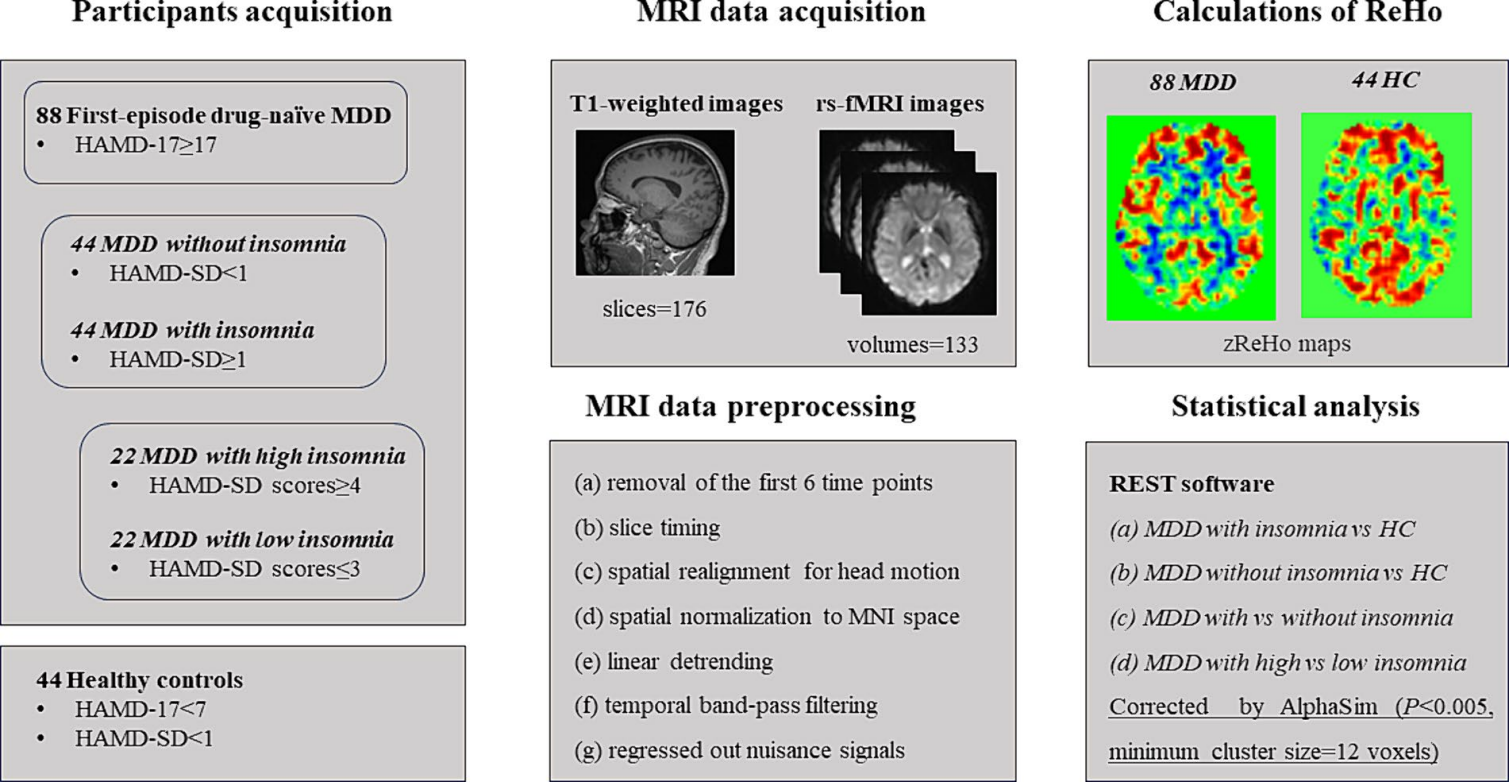
The flow chart for this study. MDD: major depressive disorder; HC: healthy controls. HAMD-17: 17-item Hamilton Depression Rating Scale; HAMD-SD: sleep disturbance subscale of HAMD-17.

This study was approved by the ethics committee of Nanjing Brain Hospital, Affiliated Hospital of Nanjing Medical University. In addition, written informed consent was acquired from each subject before participating in this study. A total of 88 first-episode drug-naïve MDD patients including 44 with insomnia (22 with high insomnia and 22 with low insomnia) and 44 without insomnia were recruited from the Department of Psychiatry, Nanjing Brain Hospital, Affiliated Hospital of Nanjing Medical University. Moreover, 44 right-handed volunteers matched for age, gender and years of education were recruited by local advertisements as HC. The details about demographic and clinical features of patients and HC were presented in Table 1 and the flow chart of this study was showed in Figure 1.

**Table 1:** Comparisons of demographic and clinical data between groups.

	MDD with insomnia	MDD without insomnia	HC	*F*/*χ^2^*	*P*
Gender (male/female)	22/22^d^	25/19	23/21	0.43	0.81
Age (years)	30.07 ± 6.46^d^	30.98 ± 6.51	30.80 ± 4.80	0.29	0.75
Educational level (years)	13.86 ± 2.40^d^	14.43 ± 2.02	14.57 ± 1.73	1.44	0.24
HAMD-17 (scores)	27.14 ± 3.96^e^	19.73 ± 2.18	1.68 ± 0.96	1060.47	<0.01^a^
HAMD-SD (scores)	3.50 ± 1.27^e^	0.00 ± 0.00	0.00 ± 0.00	335.90	<0.01^b^
adjusted HAMD-17 (scores)	20.14 ± 3.13^d^	19.73 ± 2.18	1.68 ± 0.96	947.31	<0.01^c^

MDD: major depressive disorder; HC: healthy controls. HAMD-17: 17-item Hamilton Depression Rating Scale; HAMD-SD: sleep disturbance subscale of HAMD-17; adjusted HAMD-17: HAMD-17 scores after omission of HAMD-SD scores.

a Higher HAMD-17 scores in MDD patients with and without insomnia when compared to HC and higher HAMD-17 scores in MDD patients with insomnia when compared to those without insomnia.

b Higher HAMD-SD scores in MDD patients with insomnia when compared to those without insomnia and H.

c Higher adjusted HAMD-17 scores in MDD patients with and without insomnia when compared to HC.

d No differences in the gender, age, educational level and adjusted HAMD-17 scores between MDD patients with high and low insomnia.

e Higher HAMD-17 and HAMD-SD scores in MDD patients with high insomnia when compared to those with low insomnia. *p* values were obtained by one-way analysis of variance and post hoc contrasts with the method of least-significant difference (age, educational level and HAMD sores), as well as Pearson chi-square test (gender). *P* < 0.05 were considered to be statistically significant differences.

The inclusion criteria for MDD patients and HC were as follows: (a) Han nationality; (b) right-handed; (c) age between 20 and 45 years; (d) educational level ≥ 9 years. All patients were diagnosed as MDD by two experienced psychiatrists according to the Diagnostic and Statistical Manual of Mental Disorders-fifth Edition (DSM-5) criteria. First-episode patients with no previous medication history were enrolled in this study if the total scores of 17-item Hamilton Depression Rating Scale (HAMD-17) were ≥ 17 and the course of MDD ranged from 3 to 24 months. Patients were categorized into the group with insomnia if they had depressive symptoms as major symptom with insomnia as concomitant symptoms, lasting for 3 months or more and the scores sleep disturbance subscale of HAMD-17 (HAMD-SD) were ≥ 1. In addition, patients with HAMD-SD scores≥4 were divided into MDD group with high insomnia while those with HAMD-SD scores≤3 were divided into MDD group with low insomnia. Moreover, HAMD-17 scores after omission of HAMD-SD scores (adjusted HAMD-17) were calculated to reevaluated the severity of MDD regardless of insomnia. The volunteers with no current or history of psychiatric disorders and favorable sleep quality (HAMD-17 sores<7 and HAMD-SD < 1) were enrolled in this study.

The exclusion criteria for all participants were as follows: (a) current or history of other psychiatric disorders screened through diagnostic interviews with the Structured Clinical Interview for DSM-5 Non-patient Edition by two experienced psychiatrists; (b) any serious somatic or neurological disorders; (c) abuse of alcohol or drugs; (d) brain lesions or structural brain abnormalities identified by routine MRI; (e) female who were regnant, lactating or menstruating; (f) any contraindication for MRI scans.

### 2.2 MRI data acquisition

MRI scanning was performed on a Siemens Verio 3.0 Tesla scanner (Siemens, Erlangen, Germany) at the Department of Radiology, Nanjing Brain Hospital, Affiliated Hospital of Nanjing Medical University. All subjects were instructed to minimize movement, keep eyes closed, remain awake and not think of anything during the entire MRI scans. The 3D T1-weighted data were acquired with the following parameters: repetition time (TR) = 1,900 ms; echo time (TE) = 2.48 ms; slice thickness = 1 mm; field of view (FOV) = 256 × 256 mm^2^; matrix = 256 × 256; flip angle (FA) = 9°; voxel size = 1 × 1 × 1 mm^3^; number of slices = 176; scan time = 4min18s. The rs-fMRI images were acquired using the following parameters: TR = 3,000 ms; TE = 40 ms; slice thickness = 4 mm; FOV = 240 × 240 mm^2^; matrix = 64 × 64; FA = 90°; voxel size = 3.8 × 3.8 × 4 mm^3^; number of slices = 32; number of volumes = 133; scan time = 6min45s.

### 2.3 MRI data preprocessing

MRI data preprocessing was performed using the software of Data Processing Assistant for Resting-State fMRI (DPARSF) advanced edition (Chao-Gan and Yu-Feng, 2010), a part of the Data Processing and Analysis for Brain Imaging (DPABI) toolbox, running in MATLAB. The main steps were referred to previous studies of our and other teams (Cao et al., 2024; Hu et al., 2024; Zhao et al., 2023; Huang et al., 2024), which were as follows: (a) removal of the first 6 time points for magnetization stabilization; (b) slice timing alignment for acquisition time delay between slices; (c) spatial realignment for head motion correction and subjects with translational motion exceeding 2 mm or rotational motion exceeding 2° were excluded; (d) spatial normalization to the Montreal Neurological Institute (MNI) space provided by SPM (voxel size: 3 × 3 × 3 mm^3^) for spatial normalization, and re-sample into a voxel size of 3 mm × 3 mm × 3 mm to align spatially with the MNI space by using new segment to the structural images (also aimed to reduce unwanted resolution loss and reduce voxel grid spacing through resampling); (e) linear detrending to reduce the influence of MRI equipment; (f) temporal band-pass filtering at a frequency band of 0.01–0.1 Hz, to reduce low-frequency drift, physiological high-frequency respiratory and cardiac noise; (g) regressed out nuisance signals (the head motion parameters, white matter and cerebrospinal fluid signals) with the Friston 24-parameter model, to control the potential impact of physiological artifacts.

### 2.4 Calculations of ReHo

After fMRI data preprocessing, individual’s ReHo values were calculated within the whole-brain mask by the software of DPARSF (Chao-Gan and Yu-Feng, 2010) based on MATLAB according to previous studies of our and other teams (Hu et al., 2024; Zhao et al., 2023; Huang et al., 2024). ReHo values are used to measure the functional synchronization of spontaneous neural activity between a voxel and its neighboring voxels, which reflect the temporal homogeneity of the regional BOLD signals and the temporal homogeneity of neural activity. Based on non-smooth rs-fMRI data, ReHo values (also called Kendall’s coefficient of concordance, KCC) were calculated by the synchronization of the time series of a given voxel with those of its 26 adjacent neighbors (26 voxels) in a voxel-wise analysis, and then ReHo maps were generated for all individuals. To improve the normality of ReHo values across subjects, ReHo values were standardized by *Fisher’s r-to-z* transformation and then zReHo maps of the whole brain were obtained for all subjects. Finally, to reduce the artifacts and biases that affect analysis accuracy, smoothing was used to improve signal-to-noise ratio (Wang J. et al., 2022), and all zReHo maps were spatially smoothed with a 4 mm full-width-at-half-maximum (FWHM) Gaussian kernel, generating the final smoothed zReHo (szReHo) maps.

### 2.5 Statistical analysis

Between-groups differences of demographic and clinical characteristics were evaluated by one-way analysis of variance (ANOVA) and *post hoc* contrasts using the software of Statistical Package for the Social Sciences (SPSS). *p* < 0.05 was considered to be statistically significant differences. In addition, ANOVA was performed to evaluate differences in ReHo values among three groups, and *post hoc t*-tests were used to identify between-groups differences of ReHo values with the software of Resting-State fMRI Data Analysis Toolkit (REST)(Song et al., 2011). The significant differences were set at *p* < 0.005 (voxel-level) with an additional requirement set for the minimum cluster size of 12 voxels, which were corrected for multiple comparisons by the AlphaSim program in REST software, the actually estimated FWHM was 4 mm. Moreover, receiver operating characteristic (ROC) curves were conducted to determine the values of abnormal brain regions for identifying MDD patients with insomnia and evaluating the severity of insomnia. The significant level was set at *p* < 0.05.

## 3 Results

### 3.1 Comparisons of demographic and clinical data between groups

There were no significant differences in the gender, age and educational level between groups of MDD patients with insomnia, MDD patients without insomnia and HC. Higher HAMD-17 scores were found in MDD patients with and without insomnia when compared to HC. Higher HAMD-17 scores were detected in MDD patients with insomnia when compared to those without insomnia. However, no significant differences were found in the adjusted HAMD-17 scores of MDD patients with and without insomnia, which were significantly higher than that of HC. MDD patients with insomnia demonstrated higher HAMD-SD scores when compared to those without insomnia and HC. No significant differences were revealed between MDD patients without insomnia and HC (Table 1).

In addition, there were no significant differences in the gender, age and educational level between groups of MDD patients with high and low insomnia. In comparison with HC, both MDD patients with high and low insomnia exhibited higher HAMD-17 scores. MDD patients with high insomnia had higher HAMD-17 compared to those with low insomnia. However, no differences were found in adjusted HAMD-17 scores between MDD patients with high and low insomnia. MDD patients with high insomnia had higher HAMD-SD scores than that of those with low insomnia (Table 1).

### 3.2 Brain regions showed differences of ReHo values

#### 3.2.1 Comparison of three group

**Figure 2:**
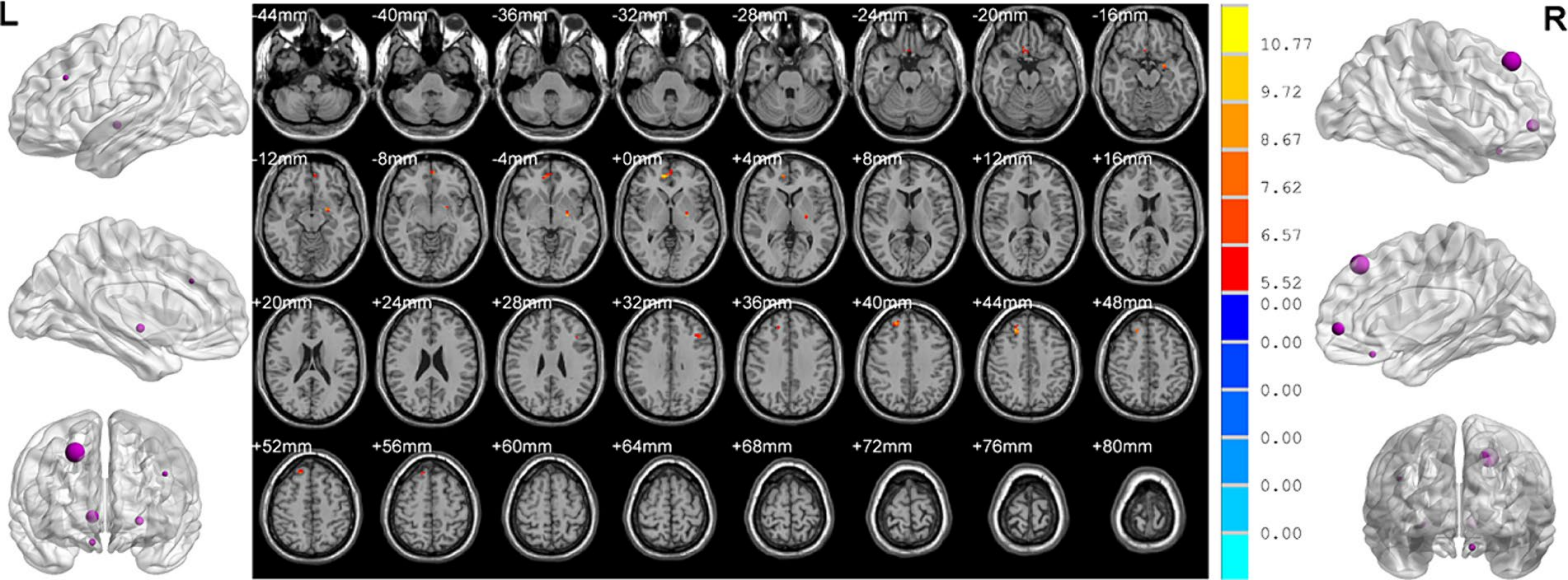
Brain regions showed differences of ReHo values among three groups. ReHo: regional homogeneity. The significant differences were set at *p* < 0.005 with an additional requirement set for the minimum cluster size of 12 voxels, which were corrected for multiple comparisons at the cluster level by the AlphaSim program in REST software.

Analysis of variance showed that there were significant differences in ReHo values in the left middle frontal gyrus, left pallidum, right superior frontal gyrus, right medial superior frontal gyrus and right rectus gyrus among three groups (Table 2; Figure 2).

**Table 2:** Brain regions showed differences of ReHo values.

	Peak MNI coordinates			Clusters	Peak *F*/*T* values
	*x*	*y*	*z*		
Comparison of three groups (ANOVA)					
Left middle frontal gyrus	−42	27	30	12	9.74
Left pallidum	−24	−9	−3	19	9.85
Right superior frontal gyrus	21	36	45	43	10.00
Right medial superior frontal gyrus	9	51	0	28	11.83
Right rectus gyrus	9	27	−18	15	7.50
MDD with insomnia vs HC (*Post hoc t*-test)					
Left medial superior frontal gyrus	−3	51	30	12	3.76
Left middle frontal gyrus	−24	45	36	36	4.01
Left triangular inferior frontal gyrus	−42	27	30	19	4.44
Left calcarine fissure	−12	−54	12	12	3.65
Right medial superior frontal gyrus	6	39	54	75	4.94
Right medial orbital superior frontal gyrus	9	51	0	76	5.02
Left middle occipital gyrus	−30	−63	33	14	−5.09
Left pallidum	−24	−9	−3	27	−4.27
Right superior temporal gyrus	60	0	−6	27	−3.83
Right inferior temporal gyrus	39	0	−33	12	−4.20
Right middle cingulate gyrus	12	3	45	24	−4.41
Right hippocampus	36	−9	−18	15	−4.10
Right putamen	30	9	0	26	−4.25
MDD without insomnia vs HC (*Post hoc t*-test)					
Left middle frontal gyrus	−45	27	33	17	3.67
Left orbital middle frontal gyrus	−21	36	−18	20	4.32
Left anterior cingulate gyrus	−6	51	0	13	4.05
Right triangular inferior frontal gyrus	42	27	21	15	4.08
Left rectus gyrus	−9	21	−18	12	−3.72
Left postcentral gyrus	−45	−30	57	25	−4.83
Right rectus gyrus	9	27	−18	28	−3.88
Right fusiform gyrus	33	0	−33	15	−3.51
Right pallidum	24	−6	−3	12	−4.27
MDD with vs without insomnia (*Post hoc t*-test)					
Left insula	−33	0	−3	12	−4.35
MDD with high vs low insomnia (*Post hoc t*-test)					
Right middle temporal gyrus	54	−57	6	12	4.01
Left orbital superior frontal gyrus	−21	60	−6	13	−4.54
Left lingual gyrus	−12	−54	3	15	−4.09
Right inferior parietal gyrus	36	−51	45	16	−4.01
Right postcentral gyrus	63	−3	36	17	−4.26

MDD: major depressive disorder; HC: healthy controls. ReHo: regional homogeneity. ANOVA: analysis of variance. MNI: Montreal Neurological Institute; x, y and z: coordinates of peak voxels of clusters in MNI space. The significant differences were set at *P* < 0.005 with an additional requirement set for the minimum cluster size of 12 voxels, which were corrected for multiple comparisons at the cluster level by the AlphaSim program in REST software.

#### 3.2.2 MDD with insomnia vs HC

**Figure 3:**
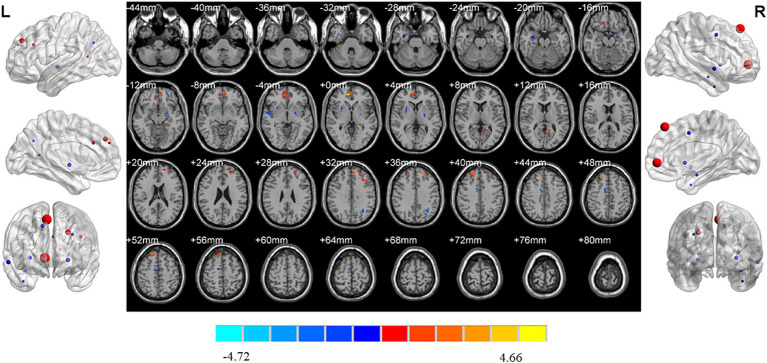
Brain regions showed differences of ReHo values between MDD patients with insomnia and HC. MDD: major depressive disorder; HC: healthy controls. ReHo: regional homogeneity. The significant differences were set at *p* < 0.005 with an additional requirement set for the minimum cluster size of 12 voxels, which were corrected for multiple comparisons at the cluster level by the AlphaSim program in REST software.

MDD patients with insomnia showed increased ReHo values in the medial superior frontal gyrus, middle frontal gyrus, triangular inferior frontal gyrus, calcarine fissure and right medial superior frontal gyrus, medial orbital superior frontal gyrus, as well as decreased ReHo values in the left middle occipital gyrus, pallidum and right superior temporal gyrus, inferior temporal gyrus, middle cingulate gyrus, hippocampus, putamen when compared with HC (Table 2; Figure 3).

#### 3.2.3 MDD without insomnia vs HC

**Figure 4:**
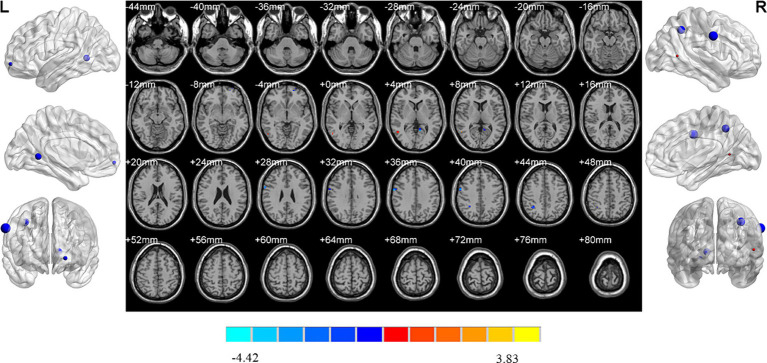
Brain regions showed differences of ReHo values between MDD patients without insomnia and HC. MDD: major depressive disorder; HC: healthy controls. ReHo: regional homogeneity. The significant differences were set at *p* < 0.005 with an additional requirement set for the minimum cluster size of 12 voxels, which were corrected for multiple comparisons at the cluster level by the AlphaSim program in REST software.

Compared with HC, increased ReHo values were detected in the left middle frontal gyrus, orbital middle frontal gyrus, anterior cingulate gyrus and right triangular inferior frontal gyrus of MDD patients without insomnia. In addition, MDD patients without insomnia demonstrated decreased ReHo values in the left rectus gyrus, postcentral gyrus and right rectus gyrus, fusiform gyrus and pallidum compared to HC (Table 2; Figure 4).

#### 3.2.4 MDD with vs without insomnia

**Figure 5:**
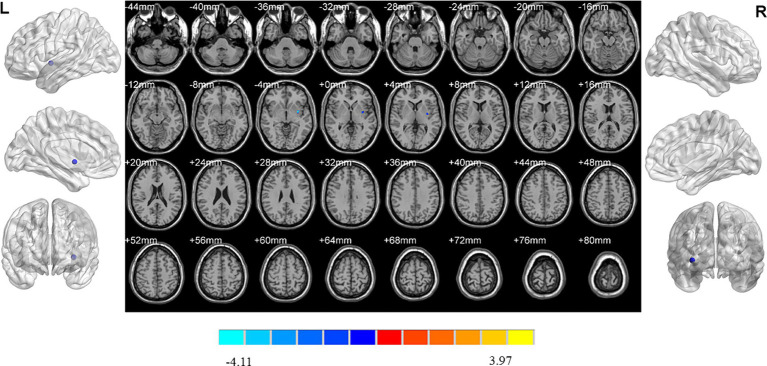
Brain regions showed differences of ReHo values between MDD patients with and without insomnia. MDD: major depressive disorder. ReHo: regional homogeneity. The significant differences were set at *p* < 0.005 with an additional requirement set for the minimum cluster size of 12 voxels, which were corrected for multiple comparisons at the cluster level by the AlphaSim program in REST software.

MDD patients with insomnia had decreased ReHo values in the left insula when compared to those without insomnia (Table 2; Figure 5).

#### 3.2.5 MDD with high vs low insomnia

**Figure 6:**
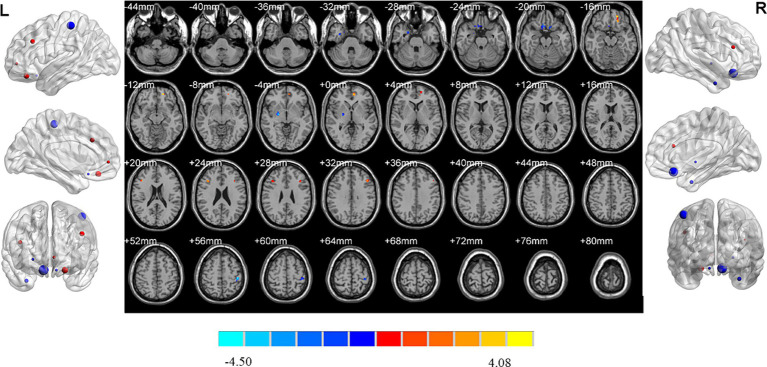
Brain regions showed differences of ReHo values between MDD patients with high and low insomnia. MDD: major depressive disorder; HC: healthy controls. ReHo: regional homogeneity. The significant differences were set at *p* < 0.005 with an additional requirement set for the minimum cluster size of 12 voxels, which were corrected for multiple comparisons at the cluster level by the AlphaSim program in REST software.

MDD patients with high insomnia showed increased ReHo values in the right middle temporal gyrus, and decreased ReHo values in the left orbital superior frontal gyrus, lingual gyrus, right inferior parietal gyrus and postcentral gyrus when compared with those with low insomnia (Table 2; Figure 6).

### 3.3 ROC analysis of the values of abnormal brain regions

**Figure 7:**
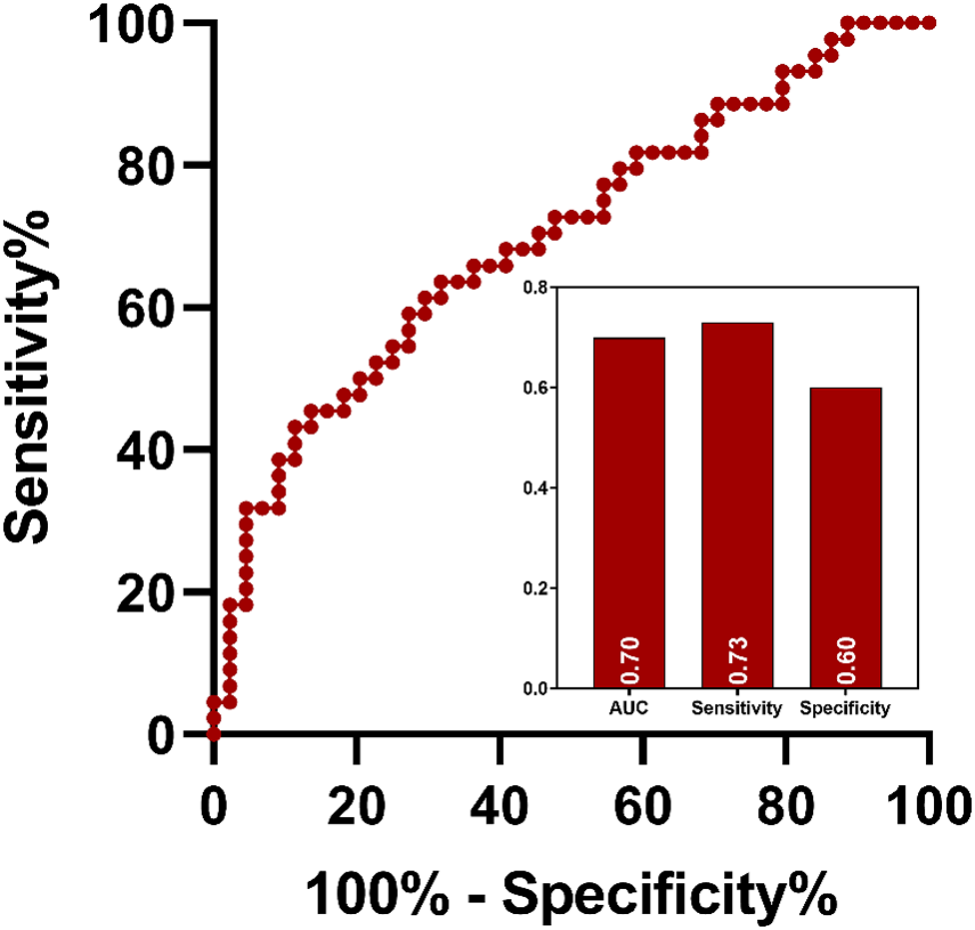
ROC analysis of the value of abnormal brain region in distinguish MDD patients with insomnia from those without insomnia. MDD: major depressive disorder; ROC: receiver operating characteristic; AUC: area under curve.

**Figure 8:**
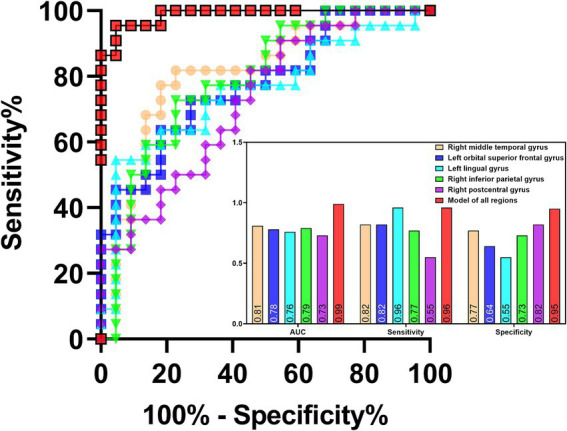
ROC analysis of the values of abnormal brain regions in evaluating the severity of insomnia in MDD patients. MDD: major depressive disorder; ROC: receiver operating characteristic; AUC: area under curve.

ROC analysis indicated that ReHo values in the left insula (AUC = 0.70, sensitivity = 73%, specificity = 60%; Figure 7) could effectively distinguish MDD patients with insomnia from those without insomnia. In addition, ReHo values of the right middle temporal gyrus (AUC = 0.81, sensitivity = 82%, specificity = 77%), left orbital superior frontal gyrus (AUC = 0.78, sensitivity = 82%, specificity = 64%), lingual gyrus (AUC = 0.76, sensitivity = 96%, specificity = 55%), right inferior parietal gyrus (AUC = 0.79, sensitivity = 77%, specificity = 73%), postcentral gyrus (AUC = 0.73, sensitivity = 55%, specificity = 82%) and the combined model (AUC = 0.99, sensitivity = 96%, specificity = 95%) were all able to evaluate the severity of insomnia in MDD patients (Figure 8).

## 4 Discussion

Based on rs-fMRI data, the measure of ReHo was utilized to evaluate the changes of brain activity of patients during rest. Analysis of variance showed that there were significant differences in ReHo values in the left middle frontal gyrus, left pallidum, right superior frontal gyrus, right medial superior frontal gyrus and right rectus gyrus among three groups. Compared with HC, MDD patients without insomnia exhibited increased ReHo values in the prefrontal regions and decreased ReHo values in the limbic system. Patients with insomnia demonstrated wider abnormal ReHo values in the prefrontal-limbic circuits, which was characterized by compensatory increased activity in the prefrontal regions and decreased activity of regions located in the limbic system. In addition, differences of ReHo values were identified in the left insula between patients with and without insomnia. Moreover, high level of insomnia might be associated with the decreased ReHo values of brain regions within the frontal–parietal network. Finally, ROC analysis demonstrated that impaired brain region might be helpful for identifying MDD patients with insomnia and evaluating the severity of insomnia.

Based on distinctive imaging approaches, objective differences in brain regions and changes in neural activity were found in patients with MDD and insomnia individuals in previous neuroimaging studies (Kaiser et al., 2015; Wang et al., 2020; Zhu et al., 2021; Liu et al., 2016). In addition, these neuroimaging features could be used as biomarkers for reflecting individual sleep disturbances and diagnosing MDD (Shi et al., 2021). Moreover, changes in the activity of some brain regions, such as the salience network, the suprachiasmatic nuclei, and the DMN, were also identified in patients with MDD with insomnia using rs-fMRI (Liu et al., 2018; McKinnon et al., 2018; Gong et al., 2021). Sleep electroencephalography spectral power was found to be associated with subjective-objective discrepancy of sleep onset latency in patients MDD (Kang et al., 2018). Electrophysiological brain activity during sleep was also found to be related to increased suicidal ideation in MDD patients, which suggested that central nervous system hyperarousal during sleep might be a neurobiological correlate of suicidal ideation (Dolsen et al., 2017). However, these studies mentioned above did not remove the interferences of drugs.

In this study, MDD patients showed decreased activity in the prefrontal regions and increased activation in the subcortical structures including pallidum, putamen and hippocampus when compared with HC. In addition, differences of brain activity were detected in the insula between MDD patients with and without insomnia. These findings were consistent with findings of previous neuroimaging study (Lu et al., 2021; Qin et al., 2021; He et al., 2019). The prefrontal cortex is regard as a vital region implicated in the integration of emotional information and the regulation of the intensity of emotional responses while the limbic and subcortical regions are responsible for the emotional processes (Dixon et al., 2017; Rolls, 2015). The prefrontal-limbic-subcortical network is involved in the regulation of emotion and it is considered as an important circuitry in the pathophysiology underlying depression (Lu et al., 2021). Disrupted functional connectivity were identified in the cortical–limbic system, frontoparietal network, frontal-insula system, as well as between DMN and limbic systems in patients during depressive episodes (Magioncalda et al., 2015; Goya-Maldonado et al., 2016; Yin et al., 2018; Wang et al., 2017). Functional abnormalities in the prefrontal-limbic-subcortical system were associated with the impaired emotion and affective processing, which was the core feature of patients with depression and might serve as a promising biomarker for depression (Radaelli et al., 2015). Both bottom-up dysfunction and disrupted top-down emotion regulation were identified in depressive patients, which represented by abnormal neural activation in the limbic and subcortical structures, as well as in altered neural activation in the prefrontal regions (Phillips et al., 2008). Altered functional connectivity was found between the prefrontal cortex and striatum including putamen and caudate, as well as between the prefrontal cortex and limbic including insula in patients during depressive episodes (Lu et al., 2021). Abnormal brain activities within the prefrontal-limbic-striatal circuits were highly associated with mood dysregulation and might be a candidate reliable biomarker for depression, which might be the neurological basis underlying depressive emotion (He et al., 2019). Therefore, abnormal activation of the frontal-limbic-striatal circuits might cause impaired bottom-up emotional processing and failure top-down emotional regulation (Graham et al., 2013; Ochsner et al., 2012), which resulted in emotional dysregulation and insomnia in MDD patients with insomnia.

Previous neuroimaging study showed that the features of spontaneous brain activities could be used as potentially for distinguishing patients with insomnia from HC based on rs-fMRI data (Yang et al., 2023). Previous rs-fMRI study showed that patients with insomnia had decreased spontaneous regional brain activity in the middle, inferior frontal gyrus and inferior parietal lobule, which were negatively correlated with the duration of and severity of insomnia (Li et al., 2016). Increased ReHo values of the left fusiform gyrus and decreased ReHo values of the bilateral cingulate gyrus were revealed in patients with insomnia, which might be the neural mechanisms underlying negative emotion and insomnia (Dai et al., 2014). Abnormal ReHo values were also found in the insula, anterior cingulate gyrus, precentral gyrus, cuneus, middle cingulate cortex and fusiform of insomnia patients, and these abnormal spontaneous activities were mainly located in the emotion-related circuits (Wang et al., 2016). Decreased brain activities were detected in brain regions involved in alertness, attention and higher-order cognitive processes and increased activities were found in auditory-related and vision-related regions of insomnia patients, which might be associated with the hyperarousal state and the loss of inhibition in sensory-informational processing (Zhou et al., 2017; Li et al., 2016). Abnormal local activities in the visual processing-related, sensorimotor cortex and DMN might be also related to insomnia and the over-arousal of DMN might be the main pathological mechanism underlying insomnia leading to emotion dysregulation and cognitive impairments (Wang H. et al., 2022).

In this study, decreased activity of the insula might be associated with occurrence of insomnia in MDD patients. Decreased activities of the frontal–parietal network might be related to the severity of insomnia in MDD patients. These findings were in accordance with previous neuroimaging studies. The insula is considered as a key region of the salience network, which is involved in modulating activities of the executive control network and DMN for better cognitive performance (Drummond et al., 2013). Aberrant functional connectivity was identified between the insula and prefrontal regions, which might be the candidate substrate for cognitive impairment of insomnia patients (Li et al., 2018). Additionally, insomnia patients demonstrated impaired connectivity within the frontal, parietal, temporal and subcortical regions (Wei et al., 2019). Abnormal activities in the inferior frontal gyrus and insula of the salience network were speculated to be specifically associated with the hyperarousal state of insomnia of MDD patients, independently of the effects of emotion (Liu et al., 2018). In addition, impaired functional connectivity was identified between the parietal and prefrontal cortices and the anterior cingulate gyrus of insomnia patients, which might lead to disrupted attention and working memory (Li et al., 2014). Moreover, insomnia patients showed abnormal functional connectivity in the emotion circuit including decreased functional connectivity between the amygdala and insula, striatum and thalamus, and increased functional connectivity between the amygdala and premotor cortex, sensorimotor cortex, which suggested that dysfunction of the emotional circuit might be associated with insomnia (Huang et al., 2012). Increased functional connectivity of the amygdala might be a compensatory neurobiological mechanism to overcome the condition of insomnia and related negative emotion of patients (Huang et al., 2012). The shared pathophysiology might be associated with functional abnormalities of the prefrontal, anterior cingulate cortex, amygdala and insula, and altered functional connectivity within and between the salience network and DMN might be the central neural mechanisms underlying the links between MDD and insomnia (Bagherzadeh-Azbari et al., 2019).

In this study, all rs-fMRI data were collected from a Siemens 3.0 Tesla scanner (Verio) with the following parameters: TR = 3,000 ms; TE = 40 ms. TR is a crucial parameter in fMRI research, which may have an impact on the quality of imaging. However, there is currently no exact definition for the selection of TR parameter. The minimum TR achievable in whole-head fMRI scans using conventional echo-planar imaging (EPI) is 2 s or greater (Tong et al., 2014). Generally, routine GRE EPI is used for rs-fMRI acquisition, and the TR time is generally between 2,000 ms and 3,000 ms. Previous study showed that in the high-TR dataset (2,000 ms), using the same preprocessing pipeline as in the low-TR dataset (333 ms), similar activation peaks as with the low-TR dataset could be seen (Boubela et al., 2015). In this case, if we want to improve spatial resolution and obtain more precise BOLD images, we need to sacrifice temporal resolution. Improving temporal resolution, such as reducing TR to within 1,000 ms for stronger functional connectivity, requires sacrificing spatial resolution. However, recently developed multiband EPI sequences dramatically decrease the minimum TR required for a whole brain scan, without having to compromise the quality of the BOLD images (Feinberg et al., 2010; Moeller et al., 2010; Setsompop et al., 2012). Using a multiband acquisition, whole head coverage at TR = 0.4 s can be performed routinely (Tong et al., 2014). Therefore, we would compare the impact of two types of parameters [high-TR dataset (2,000-3,000 ms), and low-TR dataset (under 1,000 ms)] on the research results in the future.

However, there were several limitations in the current study. Firstly, the small sample size of this study might lead to unstable results, so further research on the issue should be conducted with a larger sample size. Secondly, this was a cross-sectional study, therefore, we were unable to establish a causal relationship. Future longitudinal studies were needed to confirm our findings and to evaluate the change of clinical characteristics of patients over time. In addition, these findings should be validated using external datasets or through cross-validation in our further studies, to increase the reliability of the results. Thirdly, this was a rs-fMRI study, therefore, the links between brain structure and function were unclear in these patients. Given recent advances in multimodal imaging techniques, future studies with larger sample size should use multimodal MRI techniques to reveal abnormities in brain structure and function to deepen our understanding of the central neural mechanisms underlying MDD patients with and without insomnia. Finally, ReHo is strictly a local measure, while functional connectivity analysis implies connectivity between regions and not only how “active” a certain brain region is, and it would be beneficial to see connectivity analysis between regions. Furthermore, considering that functionality often relies on structural foundations, we would combine functional connectivity and structural connectivity analysis to compensate for ReHo’s lack of only observing local brain activity. The functional and structural connectivity changes underlying the mechanism of depression related insomnia in the longitudinal follow-up of this batch of samples would be explored to further verify the results of this study from the perspective of the whole brain connectivity. In addition, in order to reduce the artifacts and biases that affect analysis accuracy, we would analysis data at its native resolution (4 mm) to improve signal-to-noise ratio and used updated correction methods compared to the AlphaSim method to further validate the results of the study.

## 5 Conclusion

This was the first study to explore the differences of central neural mechanisms underlying MDD patients with and without insomnia, as well as between patients with high and low level of insomnia. Findings from this study suggested that altered brain activity in the prefrontal-limbic circuits involved in emotional and cognitive regulation, might be associated with depression and insomnia in drug-naive, first-episode patients, and highlighted the vital roles of insula and frontal–parietal network in the occurrence of insomnia and its severity, respectively. All these findings provided novel insights into the pathophysiological mechanisms of MDD with insomnia. These findings suggested that insomnia could represent a symptom cluster of MDD with a distinct neurobiological underpinning, which might need personalized treatment according to its distinguishing neurobiological substrates and specific clinical symptoms.

## Data Availability

The raw data supporting the conclusions of this article will be made available by the authors, without undue reservation.
